# Assembly of (l+d)‐Tryptophan Derivatives Containing an Imidazole Group Selectively Forms a Rare Purple Ni^2+^‐Hydrogel

**DOI:** 10.1002/open.201900214

**Published:** 2019-07-29

**Authors:** Xiao‐Juan Wang, Chuan‐Wan Wei, Shu‐Qin Gao, Bo He, Ying‐Wu Lin

**Affiliations:** ^1^ School of Chemistry and Chemical Engineering University of South China Hengyang 421001 China; ^2^ Hunan Key Laboratory for the Design and Application of Actinide Complexes University of South China Hengyang 421001 China; ^3^ Laboratory of Protein Structure and Function University of South China Hengyang 421001 China

**Keywords:** Metallohydrogel, purple hydrogel, tryptophan derivative, chiral gelator, assembly

## Abstract

Design of metal‐selective hydrogels is attractive due to potential applications in materials and biological sciences. Although much progress has been made, assembly of both l‐ and d‐amino acid derivatives was less explored for design of metallohydrogels. In this study, we synthesized a facile and small tryptophan derivative containing an imidazole ligand with both l‐ and d‐ configurations (denoted as l/d‐ImW). Intriguingly, the assembly of (l+d)‐ImW gelators was found to selectively form a Ni^2+^‐hydrogel in aqueous medium at room temperature, which shows a rare purple color and exhibits excellent multi‐responsiveness. In addition to insights into the gelation mechanism, this study provides a novel approach to the design of metallohydrogels, by the assembly of (l+d)‐amino acid derivatives containing both aromatic rings and multiple metal coordination sites.

As a new class of soft materials, supramolecular hydrogels^1^ have received much attention in the past decade, due to their potential applications in materials and biological sciences, such as for medicine delivery, tissue engineering, wound healing and signal sensors, *etc*.^2^ Metallohydrogel is a pivotal branch of hydrogels that contains metal active centers.^3^ To date, although considerable progress has been achieved in metallohydrogels containing metal ions such as Cu^2+^, Fe^3+^, Zn^2+^ and Hg^2+^,3c, 3g, 4 as well as La^3+^,^5^ *etc*. it is still desirable to develop convenient approaches for the design of metal‐selective hydrogels.^6^ The formation of the 3D networks of metallohydrogels requires not only the metal‐ligand coordination interactions, but also the non‐covalent interactions, such as hydrogen bonds, π‐π stacking, vander waals force, and hydrophobic interactions.^7^ Therefore, the design of metal‐selective hydrogels should consider to make use of these interactions.

Chirality is attractive due to important roles in the medicine, sensing, catalysis and biology, and numerous soft materials and enzymes have been produced via self‐assembly of chiral molecules.^8^ The formation of some metallohydrogels is regulated by the chirality of geltors. For example, although all native amino acids are l‐configuration, we found that the combination of both l‐ and d‐amino acid derivatives (a phenylalanine derivative with a pyridyl group) triggers the formation of a Cu^2+^‐selective metallohydrogel.^9^ Based on this observation, we were further interested in construction of chiral gelators using amino acid derivatives by tuning both metal‐ligand coordination interactions and non‐covalent interactions.

In this study, we chose to synthesize both l‐ and d‐tryptophan derivatives containing an imidazole group (Figure 1a, denoted as l/d‐ImW, Scheme S1, Figures S1 and S2), by the use of indole and imidazole groups. These two chiral derivatives contain aromatic rings with multiple N atoms, as well as an acid group, which thus provide not only non‐covalent interactions such as π‐π stacking and H‐bonding interactions, but also coordination interactions for gelation. Moreover, l‐ImW and d‐ImW are small and facile molecules, and can be easily synthesized by using an environment‐friend synthetic procedure with only two simple steps. As shown herein, we found that the assembly of (l+d)‐ImW selectively forms a rare purple Ni^2+^‐hydrogel with excellent multi‐responsiveness.

**Figure 1:**
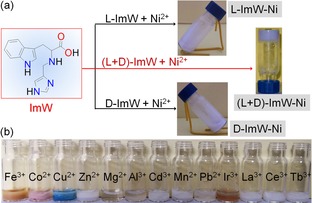
(A) The chemical structure of ImW compound, and the digital photos of gelling behaviors of Ni‐ImW complexes; (B) Digital photos of the complexes of (l+d)‐ImW and various metal ions.

The synthesized l/d‐ImW did not dissolve in pure water, but was soluble in alkaline aqueous solution. In near neutral pH values (pH 6∼8), both l‐ImW and d‐ImW gelators can rapidly self‐assemble to form a white and opaque hydrogel at room temperature, with a minimum gelling concentration of 0.04 M. For a racemic mixture of ImW ((l+d)‐ImW), the gelling process took several hours under the same conditions. Nevertheless, neither l‐ImW, d‐ImW, nor (l+d)‐ImF, can generate gels in aqueous solution when the pH was above 8 (Figure S3), suggesting crucial roles of H‐bonding interactions.

We tested the gelling ability of l‐ImW, d‐ImW and various metal ions, including Cu^2+^, Ca^2+^, Mg^2+^, Fe^2+^, Fe^3+^, Co^2+^ and Zn^2+^ *etc*, which showed that the obtained complexes presenting non‐gel status. Then, we carefully studied the gelation behavior of (l+d)‐ImW and various metal ions. The digital photos showed that addition of various metal ions resulted in either precipitate or suspension (Figure 1b). Meanwhile, Ni^2+^ ions were found to trigger the formation of metallohydrogel (denoted as (l+d)‐ImW−Ni) with a rare purple color, upon mixing of (l+d)‐ImW and Ni^2+^ aqueous solutions in a 2 : 1 ratio (Figure 1a and Figure S4). The gel formation needed only gentle shaking at room temperature, without the need of treatment such as sonication or heating. The (l+d)‐ImW−Ni gel was found to be sensitive to pH changes, whereas was stable at pH 9∼10. Note that neither l‐ImW nor d‐ImW could form gels under the same conditions, although the complexes were also in purple (Figure S4). These observations suggest that the assembly of (l+d)‐ImW can selectively response to Ni^2+^ ions, and the chirality of l/d‐ImW gelators is crucial in formation of metallohydrogel.

Moreover, as a control, we synthesized a phenylalanine derivative containing an imidazole group (denoted as ImF, Scheme S2), which possesses a structure similar to that of ImW. Meanwhile, ImF cannot generate the metallohydrogel under the same conditions, indicating that the pyrrole ring of indole group is pivotal to the gelation. In addition, we studied the effects of negative ions on gelling behaviors of (l+d)‐ImW using different nickel salts, including nickel chloride, nickel nitrate, nickel sulfate, and nickel acetate. The results showed that all different types of nickel salts could trigger the gelation, suggesting the minimal contribution of the anion in the gelation process.

To obtain the mechanical properties of (l+d)‐ImW−Ni hydrogel, we carried out rheological experiments. As shown in Figure S5, the average values of the storage modulus (G’) was always higher than the loss modulus (G’’). Both the G’ and G’’ values exhibited slight dependence on the frequency over the entire measurement range (0.1–100 Hz), which were almost equal within 600 s (Figure S6), indicating the formation of a stable gel phase. Moreover, the (l+d)‐ImW−Ni hydrogel was stable for several weeks at room temperature in a sealed container.

We further evaluated the response of (l+d)‐ImW−Ni hydrogel to several physical and chemical stimuli (Figure 2). The T_gel_ was determined to be 69±1 °C and the sol‐to‐gel transition occurred after several minutes, suggesting a thermo‐reversible property. After vigorous shaking by hand, the gel turned into a turbid liquid that restored to a self‐supported gel within several seconds, indicating a rapid thixotropic response. The (l+d)‐ImW−Ni metallohydrogel also exhibited chemical responsiveness. For example, the addition of a stoichiometric amount of solid crystalline EDTA to the gel resulted in collapse of gel and change of color from purple to glaucous within 15 min, owing to the strong binding of EDTA with nickel ions. The sol‐to‐gel transformation could be restored by an addition of excess Ni^2+^ ions. As mentioned above, the (l+d)‐ImW−Ni gel was stable at pH 9∼10. When titrated with HCl, it collapsed (pH<9) and appeared as a transparent yellowish green solution (pH<2), and turned to turbid liquid by further addition of excess NaOH (pH>10).

**Figure 2:**
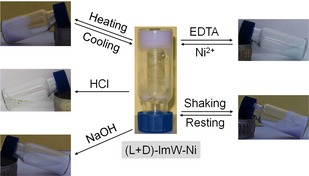
Gel‐sol transitions of the (l+d)‐ImW−Ni metallohydrogel triggered by various stimuli (mechanical, thermal, pH, and chemical reactions).

To obtain the morphological details of (l+d)‐ImW−Ni hydrogel, we carried out electronic microscopy studies. As shown by scanning electron microscopy (SEM) in Figure 3a, the gel scaffold presented as a sheet‐like structure with interconnected networks, which agrees with the transmission electron microscopy (TEM) image (Figure S7). Nevertheless, the microstructure of the (l+d)‐ImW−Ni hydrogel was different from those of the l‐ImW−Ni and d‐ImW−Ni self‐assembled into short fibers (Figs. S8b and S8c). These observations elucidated that the chirality of the gelator plays an important role in the construction of dense and entangled fiber networks, likely due to both coordination effects and the coherent effects among various non‐covalent interactions.

**Figure 3:**
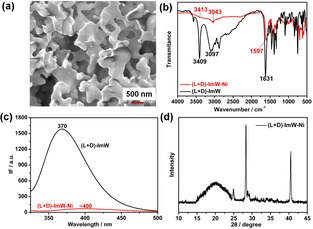
(a) SEM image of (l+d)‐MT−Ni gel, (b) FT‐IR spectra of (l+d)‐MT and (l+d)‐MT−Ni xerogel, (c) Fluorescence spectra of (l+d)‐MT and (l+d)‐MT−Ni, (d) XRD pattern of (l+d)‐MT−Ni xerogel.

To further obtain an insight into the gelling mechanism, we performed FT‐IR studies. As shown in Figure 3b, the peak at 3409 cm^−1^ (ν_−NH_) shifted to 3413 cm^−1^, and the peak 3097 cm^−1^ (ν_−COOH_) shifted to 3043 cm^−1^ in presence of Ni^2+^ ions, indicating that both amino N atoms and the carboxyl O atoms of ImW gelators are coordinated to the Ni^2+^ ions. Furthermore, the peak of imidazole ring skeleton stretching vibration at 1631 cm^−1^ shifted to 1597 cm^−1^, suggesting the involvement of the imidazole N atom in the formation of (l+d)‐ImW−Ni metallohydrogel.

The fluorescence spectra of (l+d)‐ImW and the (l+d)‐ImW−Ni complex are shown in Figure 3c. The luminescence strength of (l+d)‐ImW was reduced after addition of Ni^2+^ ions, and the peak intensity at 370 nm was quenched and shifted to 400 nm, indicating a possible energy transfer between Ni^2+^ and ImW gelator.4h Moreover, these results suggest the coordination interactions between the ImW gelator and Ni^2+^ ions.

Isothermal titration calorimetry (ITC) studies further showed that (l+d)‐ImW bound Ni^2+^ with a binding constant (*K*_a_) of 7.62×10^5^ M^−1^, with a stoichiometry close to 2 (2.11 ± 0.03) (Figure S9a), indicating a coordination ratio of 2 : 1 between (l+d)‐ImW and Ni^2+^. This result corresponded to the mass spectrometry (MS) result, which identified an ionized form of ImW−Ni complex, [Ni(ImW)_2_ ‐ H]^+^(Figure S10).

As shown in Figure 3d, the X‐Ray Diffraction (XRD) results showed a common broad diffraction peak at 20° referring to the amorphous nature of the gel. A typical peak at a d‐spacing of 2.3 Å was assigned to the strong H‐bonds that are important to form the fibrillar networks. In addition, the peak at a d‐spacing of 3.3 Å indicated strong π‐π interactions in the gelling process.^10^ Moreover, the peak at a d‐spacing of 3.2 Å (2θ=28.2°) confirmed the presence of metal‐metal interactions in the gelling process.^11^

It should be noted that although l‐ImW−Ni complex also exhibited a purple color, it presented as a purple suspension, instead of a metallohydrogel (Figure S4). Moreover, although ITC, ESI‐MS, IR, TGA and XPS results of l‐ImW−Ni complex were similar to those of (l+d)‐ImW−Ni complex (Figs. S9‐S14), their XRD patterns were different from each other (Figure S15), suggesting a different assembly mechanism. The reason that (l+d)‐ImW−Ni can generate metallohydrogel is presumably due to the suitable packing between l‐ImW and d‐ImW chiral ligands by strong π‐π interactions, as well as H‐bonds interactions.

Based on all above observations, we proposed a plausible mechanism for the assembly of l‐ImW−Ni and (l+d)‐ImW‐Nil (Scheme 1). A possible coordination approach is that l/d‐ImW binds to Ni^2+^ (2 : 1) as a tridentate ligand, forming l‐ImW−Ni and (l+d)‐MT−Ni complexes. Meanwhile, with a single chiral ligand l‐ImW, the l‐ImW−Ni complex may not self‐assemble well to form a hydrogel, due to weak non‐covalent interactions. Distinctly, for the complex of (l+d)‐MT−Ni complex, the l‐ and d‐chiral ligands presumably enable strong π‐π stacking of the indole rings, as well as H‐bonds interactions between the N atoms of indole and imidazole groups, likely bridged by water molecules, resulting in the generation of the unique Ni‐metallohydrogel.

**Scheme 1:**
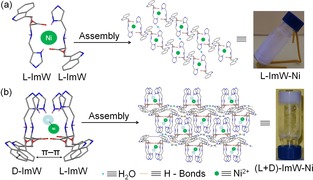
Schematic representation of the assembly of (a) L‐ImW−Ni, and (b) (l+d)‐ImW−Ni metallohydrogel.

In conclusion, we have synthesized a simple gelator of tryptophane derivative containing an imidazole group (ImW). The combination of l‐ImW and d‐ImW with a 1 : 1 ratio in presence of Ni^2+^ resulted in selective formation of a novel (l+d)‐MT−Ni metallohydrogel with a purple color, which was not observed for either l‐ImW or d‐ImW. To the best of our knowledge, this is a rare report of a metallohydrogel precursor that specifically responds to Ni^2+^ to form a purple Ni^2+^‐hydrogel. This metallohydrogel was shown to exhibit excellent multi‐responsiveness. A series of characterizations (TEM/SEM, FT‐IR, fluorescence spectroscopy, ESI‐MS, ITC and XRD) provided valuable insights into the mechanism of formation of (l+d)‐MT−Ni metallohydrogel. This study provides a novel approach to the design of metallohydrogels, by the assembly of (l+d)‐amino acid derivatives containing both aromatic rings and multiple metal coordination sites. We believe that this ingenious approach could be generally applicable to the design of other specific metallohydrogels with advanced property and function, thereby with potential applications in biological and materials sciences.

## Experimental Section

Experimental details and additional data were provided in Supporting Information.

## Conflict of interest

The authors declare no conflict of interest.

## Supporting information

As a service to our authors and readers, this journal provides supporting information supplied by the authors. Such materials are peer reviewed and may be re‐organized for online delivery, but are not copy‐edited or typeset. Technical support issues arising from supporting information (other than missing files) should be addressed to the authors.

Supplementary
