# Supplementary material for: Assembly of (l+d)‐Tryptophan Derivatives Containing an Imidazole Group Selectively Forms a Rare Purple Ni2+‐Hydrogel
Source: ChemistryOpen. 2019 Jul 29;8(9):1172–5. doi: 10.1002/open.201900214 (PMC6718073; doi:10.1002/open.201900214)
Supplement: Supplementary file 1 — Supplementary [file OPEN-8-1172-s001.pdf]

Open Access
ChemistryOPEN

Supporting Information

© Copyright Wiley-VCH Verlag GmbH & Co. KGaA, 69451 Weinheim, 2019

Assembly of (L+D)-Tryptophan Derivatives Containing an Imidazole Group Selectively Forms a Rare Purple Ni^{2+}-Hydrogel

Xiao-Juan Wang, Chuan-Wan Wei,* Shu-Qin Gao, Bo He, and Ying-Wu Lin*©2019 The Authors. Published by Wiley-VCH Verlag GmbH & Co. KGaA.
This is an open access article under the terms of the Creative Commons Attribution License, which permits use, distribution and reproduction in any medium, provided the original work is properly cited.

Supporting Information

Contents

1. Experimental Section

1.1 Reagents p.S2

1.2 Synthesis of ImW and ImF ligands p.S2

1.3 Preparation of (L+D)-ImW-Ni metallohydrogels p.S3

1.4 ITC studies p.S3

1.5 SEM, TEM, ESI-MS and NMR studies p.S4

2. Supplementary Figures

Fig. S1 ESI-MS spectrum of compound ImW. p.S4

Fig. S2 ^{1}H NMR spectrum of compound ImW in D_2O. p.S5

Fig. S3 Gelling digital photos of L-ImW, D-ImW, and (L+D)-ImW, respectively. p.S5

Fig. S4 Digital photos of the complexes of L-ImW-Ni, (L+D)-ImW-Ni, D-ImW-Ni. p.S5

Fig. S5 Dynamic frequency sweep of fresh (L+D)-ImW-Ni metallohydrogel at MGC, measured at 0.1% strain. p.S6

Fig. S6 Dynamic time sweep of fresh (L+D)-ImW-Ni metallohydrogel at MGC, measured at 0.1% strain. p.S6

Fig. S7 TEM image of (L+D)-ImW-Ni xerogel p.S7

Fig. S8 SEM image of L-ImW-Ni and D-ImW-Ni complexes. p.S7

Fig. S9 Microcalorimetric titration of (L+D)-ImW and L-ImW with Ni^{2+}. p.S7

Fig. S10 ESI-MS spectra of compound (L+D)-ImW-Ni. p.S8

Fig. S11 ESI-MS spectra of compound L-ImW-Ni. p.S8

Fig. S12 FT-IR spectra of (L+D)-ImW-Ni xerogel and L-ImW-Ni powder. p.S8

Fig. S13 TGA of (L+D)-ImW-Ni xerogel and L-ImW-Ni powder. p.S9

Fig. S14 XPS of (L+D)-ImW-Ni xeroge and L-ImW-Ni powder. p.S9

Fig. S15 XRD pattern of (L+D)-ImW-Ni xerogel and L-ImW-Ni powder. p.S10

1. Experimental Section

1.1 Reagents

Tryptophan, 1H-Imidazole-4-carbaldehyde and sodium borohydride ($NaBH_4$) were purchased from Aladin Reagent (Shanghai, China), and were used without further purification. All other reagents were of analytical grade, which include HCl, KOH, $FeCl_3\cdot 6H_2O$, $CoCl_2\cdot 5H_2O$, $Cu(Ac)_2\cdot H_2O$, $ZnSO_4\cdot 6H_2O$, $MgCl_2$, $Al_2(SO_4)_3\cdot 8H_2O$, $CaCl_2$, $CdCl_2$, $MnSO_4$, $Pb(Ac)_2$, $IrCl_3\cdot 6H_2O$, $La(NO_3)_3\cdot 6H_2O$, $CeCl_3\cdot 7H_2O$, $TbCl_3\cdot 6H_2O$, $Ni(Ac)_2\cdot 6H_2O$, $NiCl_2\cdot 6H_2O$, $Ni(NO_3)_3\cdot 6H_2O$, and $NiSO_4\cdot 6H_2O$. Deionized water (MillQ, 18.2 MΩ) was used.

1.2 Synthesis of ImW and ImF ligands

1.2.1 Synthesis of ImW ligands

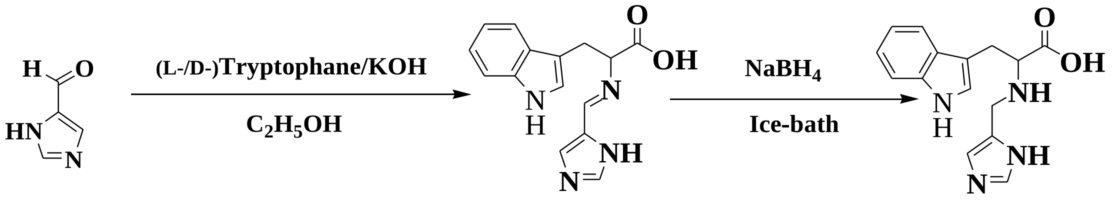

(L-/D-)ImW

Scheme S1. Synthetic route of ImW.

1.2.2 Preparation of ImW

The compound ImW was prepared following a modified literature procedure. To an aqueous solution (8 mL) of tryptophan (1 g, 5 mM) containing KOH (0.28 g, 5 mM), 1H-Imidazole-4-carbaldehyde (0.48 g, 5 mM) in CH_3CH_2OH (5 mL) was added slowly. The solution was stirred for 3 h at 40 ^{o}C. Then the solution was cooled in an ice bath. $NaBH_4$ (0.23 g, 6 mM) was added to the solution slowly. The mixture was stirred for 4 h, and 6mol$\cdot$L^{-1} hydrochloric acid was used to neutralize the basic (pH~10) reaction mixture and the pH was adjusted to 6.0-7.0. The mixture system was stirred further for 3 h. The resulting solid was filtered off, and was washed with ethanol and water, then dried. The synthetic method of L-ImW is same with that of D-ImW. Yield (L-ImW):1.06 g, 71.6%, and Yield (L-ImW): 0.98 g, 66.2%.

ESI-MS (Q-TOF): calc. for $C_{15}H_{16}N_4O_2$ 284.13, observed 285.17 $[M + H]^-$.

^1H NMR (500 MHz, D_2O, ppm): $-CH_2$ (3.11-3.13, d, 2H). -CH (3.53, 1H), $-CH_2$ (3.59, s, 2H),$-CH_2$ (3.69-3.72, d, 2H), Im-H (6.84, s, 1H), Phe-H (7.14-7.26, m, 4H), NH (7.46-7.50, 2H), Im-H (7.68-7.70, 1H).

1.2.3 Synthesis ofImF ligands

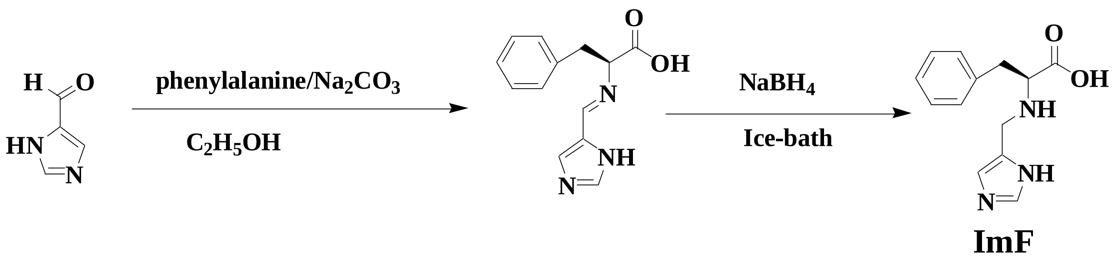

Scheme S2. Synthetic route of ImF.

1.2.4 Preparation of ImF

The procedure for preparing ImF is similar to that of ImW, except for replacing tryptophan with phenylalanine.

ESI-MS (Q-TOF): calc. for $C_{13}H_{15}N_3O_2$ 245.11, observed 244.11 [M - H]-, 282.07 [M+K - 2H]-.

^1H NMR (500 MHz, D_2O, ppm): $-CH_2$ (2.93-2.94, d, 2H). -CH (3.35-3.38, 1H),$-CH_2$ (3.67-3.88, dd, 2H), Im-H (6.87, 1H), Phe-H (7.26-7.37, m, 5H), Im-H (7.47, 1H).

1.3 Preparation of (L+D)-ImW-Nimetallohydrogels

(L+D)-ImW-Ni metallohydrogel was prepared by mixing racemic mixtures of ImW ((L+D)-ImW) solution (0.1M, ~pH 9-10) and Ni^{2+} solution (0.1M) in the ratio of 2:1 (volume). The mixture was changed into a purple metallohydrogel after shaking for several seconds.

1.4 ITC studies

Isothermal titration calorimetry (ITC) measurements were performed on a Microcal VP-ITC microcalorimeter (GE life sciences). Both (L+D)-ImW and Ni^{2+} solution were thoroughly degassed in a ThermoVal apparatus (Microcal). For titration

experiments, ~1.5 mL Ni^{2+} (0.5 mM) solution was placed in the reaction cell, and a solution of (L+D)-ImW (0.5 mM) was injected over 20 s with a total of 25 injections (10 μL for per injection), with a 150 s interval between each injection. The reaction cell was continuously stirred at 502 rpm, and heat changes were recorded at 298.15 K. The data were analyzed and the binding isotherm was fitted to a single-site model in the Origin 7.0 software (GE life sciences).

1.5SEM,TEM, ESI-MS and NMR studies

Scanning electron microscope (SEM) images were obtained on a FEI HELIOS NanoLab 600i SEM (America).Transmission electron microscope (TEM) images were obtained from a FEI Titan microscope (America). (L+D)-ImW-Ni, L-ImW-Ni complexes and ImW mass spectrum study was obtained by using Xevo G2-XS QTof mass spectrometer (Waters, America). The (L+D)-ImW-Ni, L-ImW-Ni complexes and ImW were dissolved in water and then passed through a 0.22 μm membrane filtration. NMR experiments were performed by using AMX-500 (Bruker, Switzerland).

The preparation of samples for SEM: First, a small amount of (L+D)-ImW-Ni, L-ImW-Ni, or D-ImW-Ni samples was dripped onto silicon wafers using pipetting gun, respectively. Then these samples were frozen in -80 °C refrigerator. Afterwards, they were further frozen by freeze drying machine and waiting for the SEM test.

2. Supplementary Figures

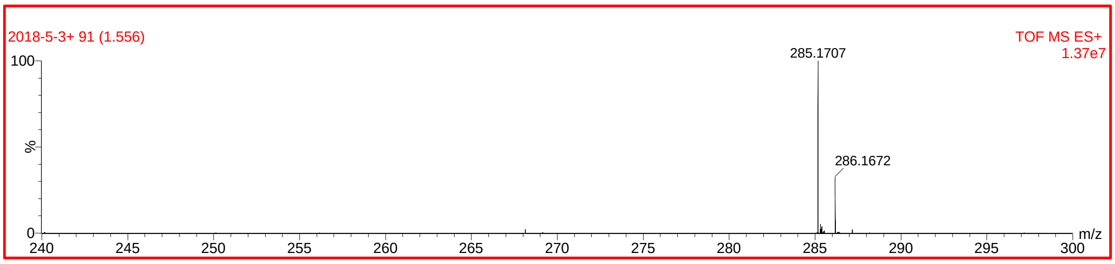

Fig. S1 ESI-MS spectrum of compound ImW, calc. for $C_{15}H_{16}N_4O_2$ 284.13, observed 285.17 $[M + H]^-$.

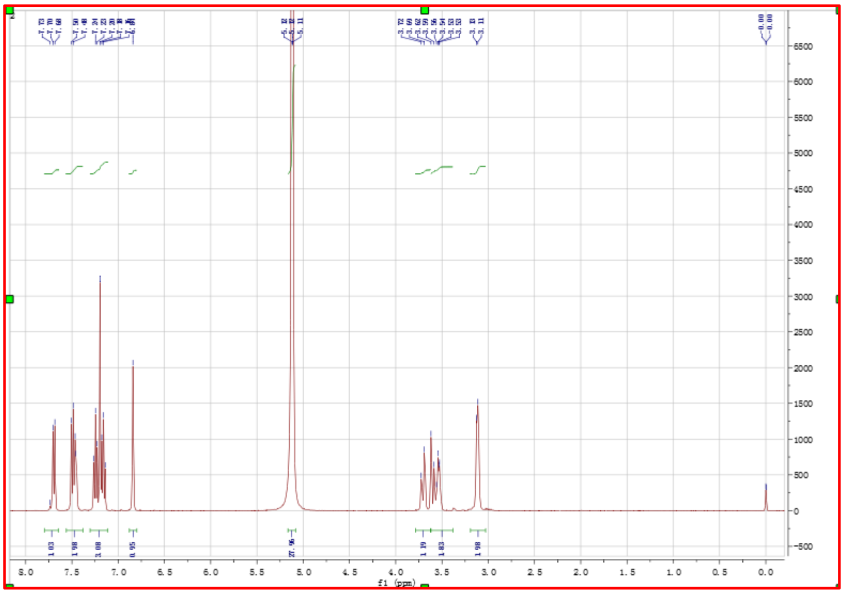

Fig. S2 ^{1}H NMR (500 MHz) spectrum of compound ImW in D_2O.

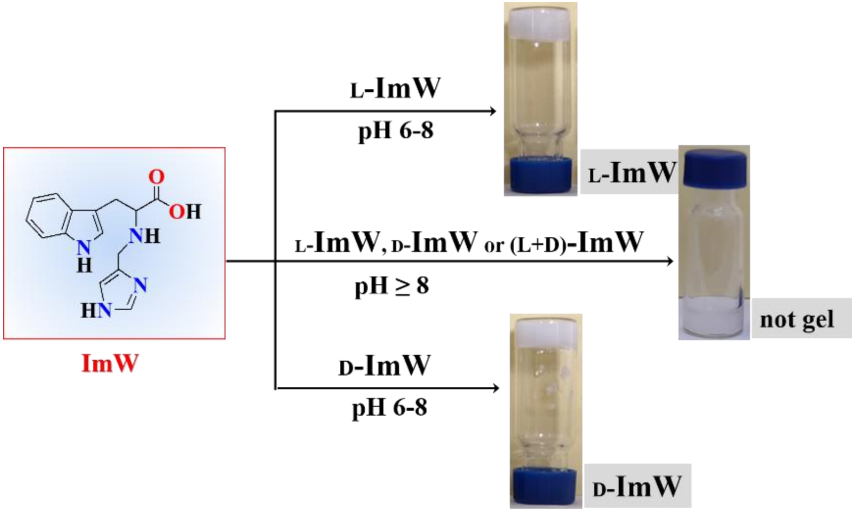

Fig. S3 Gelling digital photos of L-ImW, D-ImW, and (L+D)-ImW, respectively.

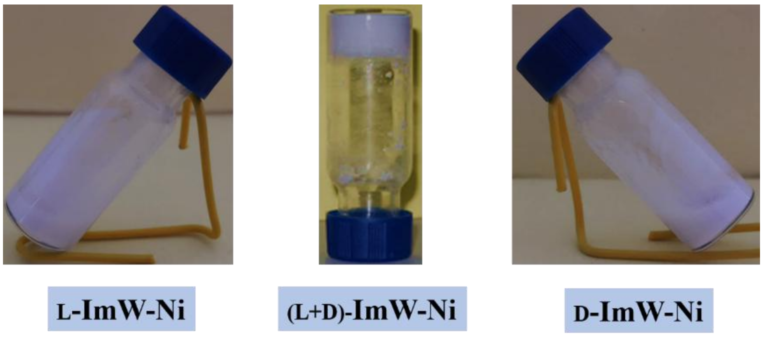

Fig. S4 Digital photos of the complexes of L-ImW-Ni, (L+D)-ImW-Ni, D-ImW-Ni, respectively.

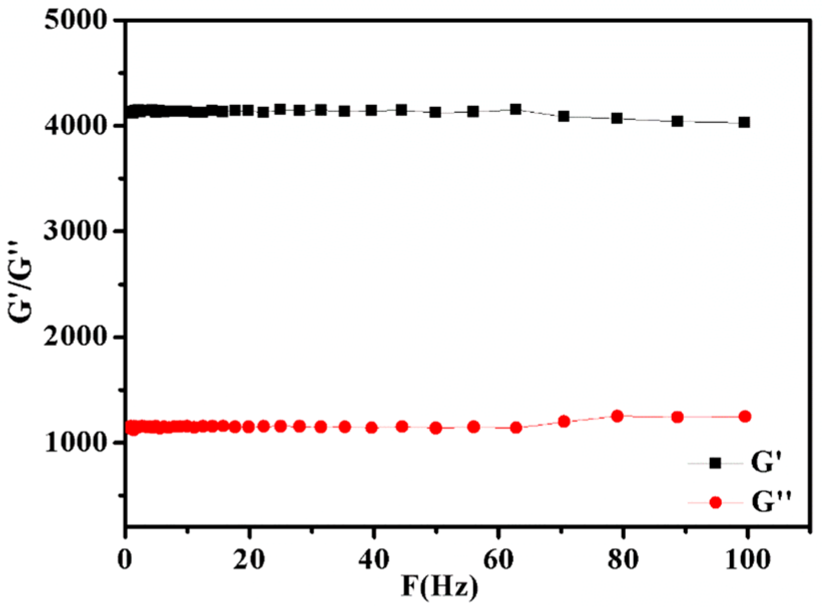

Fig. S5 Dynamic frequency sweep of fresh (L+D)-ImW-Ni metallohydrogel, measured at 0.1% strain.

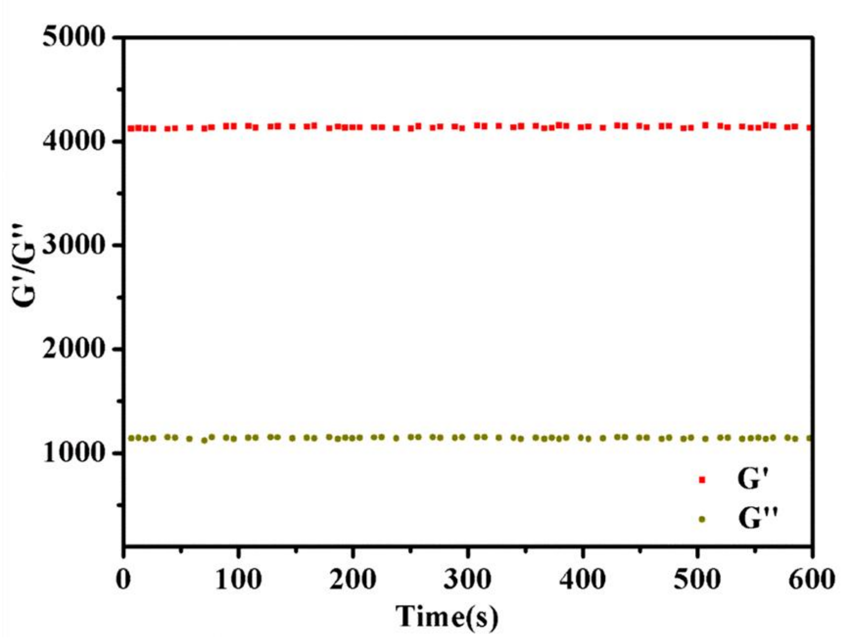

Fig. S6 Dynamic time sweep of fresh (L+D)-ImW-Ni metallohydrogel, measured at 0.1% strain.

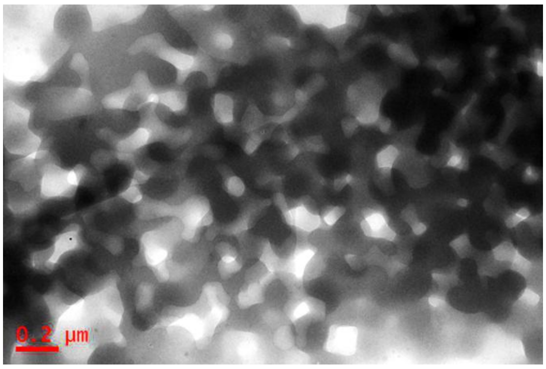

Fig. S7 TEM image of (L+D)-ImW-Ni xerogel.

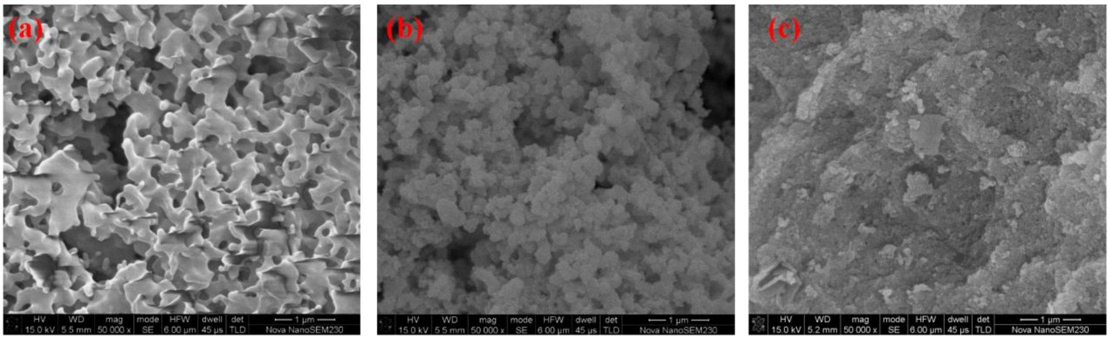

Fig. S8 SEM image of (a) (L+D)-ImW-Ni, (b) L-ImW-Ni, and (c) D-ImW-Ni complexes.

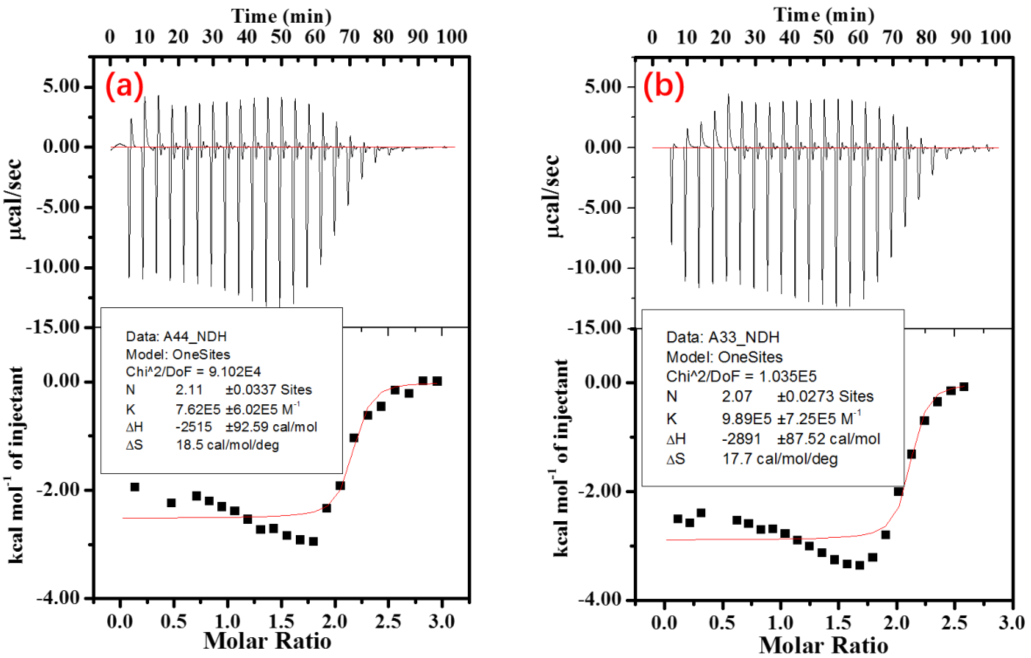

Fig. S9 Microcalorimetric titration of (a) (L+D)-ImW and (b) L-ImW with Ni^{2+} in water at 298.15 K. (Top) Raw ITC data for 25 sequential injections (10 μL per injection) of Ni^{2+} solution (8.0 mM) into ImW solution (0.5 mM). (Bottom) Net reaction heat obtained from the integration of the calorimetric traces.

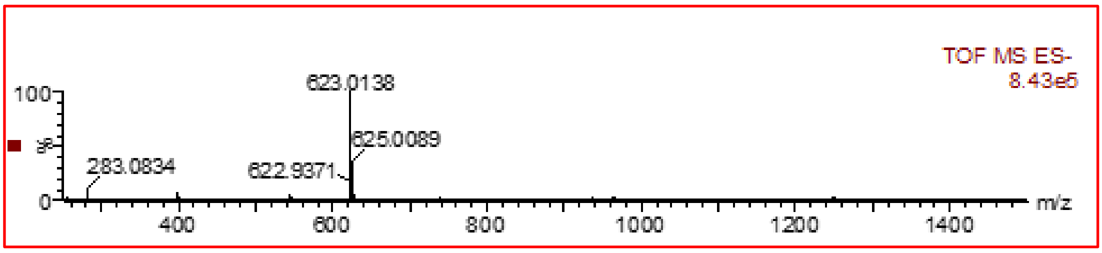

Fig. S10 ESI-MS spectra of compound (L+D)-ImW-Ni, Calculated for $C_{30}H_{30}N_8NiO_4$: 624.2 Da; Observed: 623.01 Da ($[ImW\text{-}Ni\text{-} H]^+$).

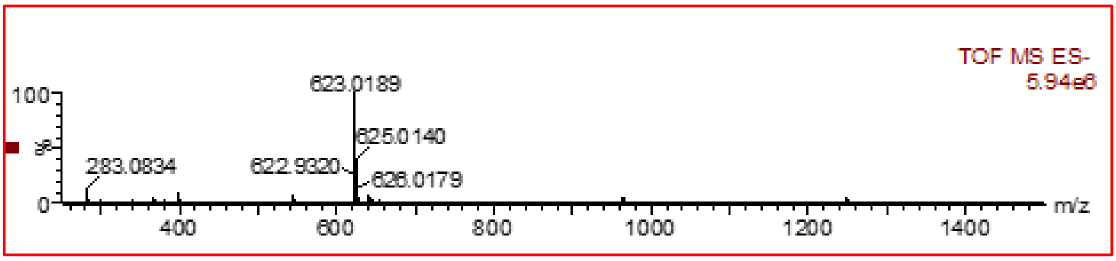

Fig. S11 ESI-MS spectra of compound L-ImW-Ni, Calculated for $C_{30}H_{30}N_8NiO_4$: 624.2 Da; Observed: 623.01 Da ($[ImW - Ni\text{-} H]^+$).

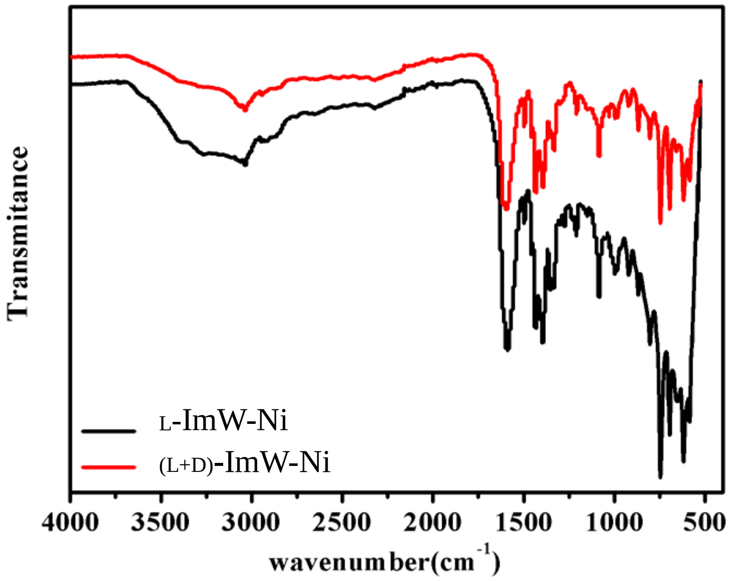

Fig. S12 FT-IR spectra of (L+D)-ImW-Ni xerogel and L-ImW-Ni powder.

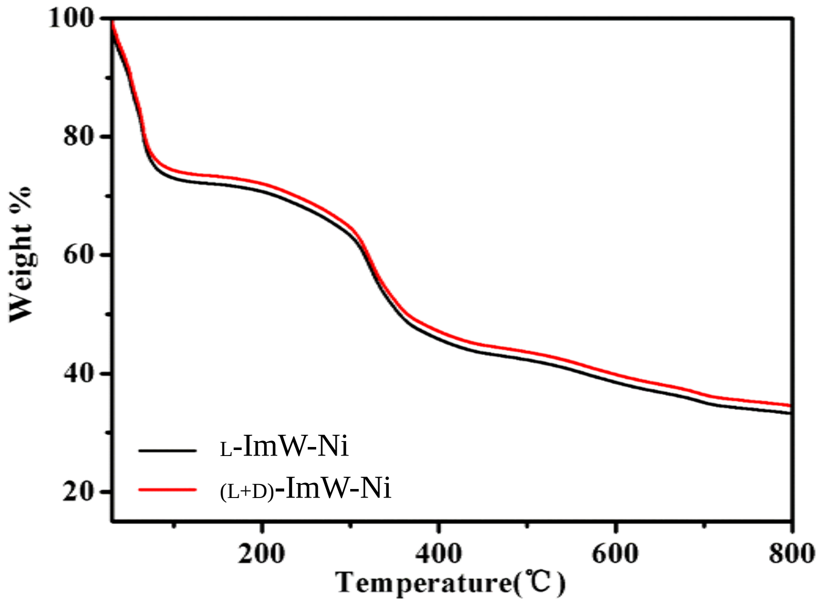

Fig. S13 Thermogravimetric analysis (TGA) thermogram of (L+D)-ImW-Ni xerogel and L-ImW-Ni powder. Thermal analysis system in a dynamic nitrogen atmosphere (heating rate: 10 ℃/min, MT-Ni, mass 1-3 mg, temperature range from room temperature up to 800 ℃).

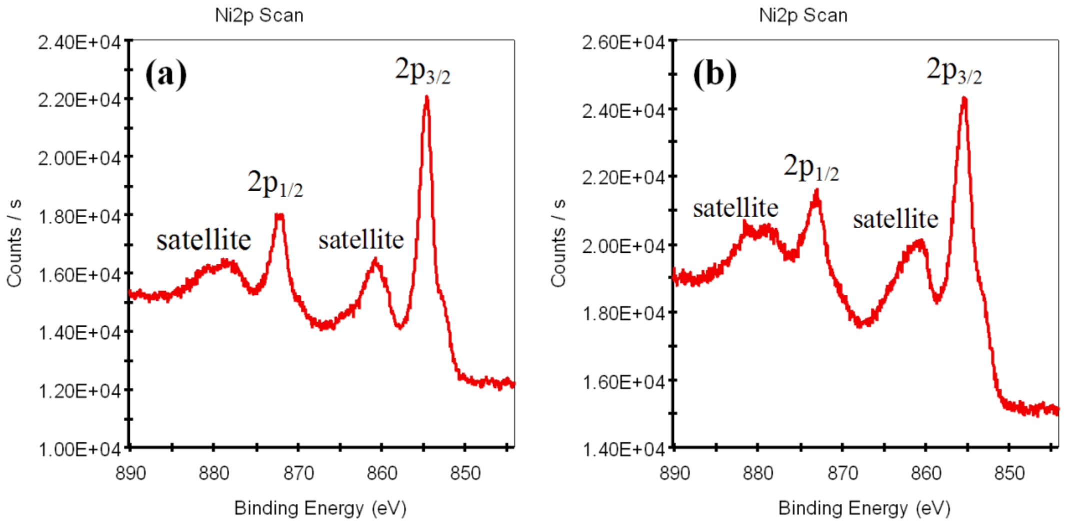

Fig. S14 X-Ray photoelectron spectroscopy (XPS) of (a) (L+D)-ImW-Ni xeroge and (b) L-ImW-Ni powder.

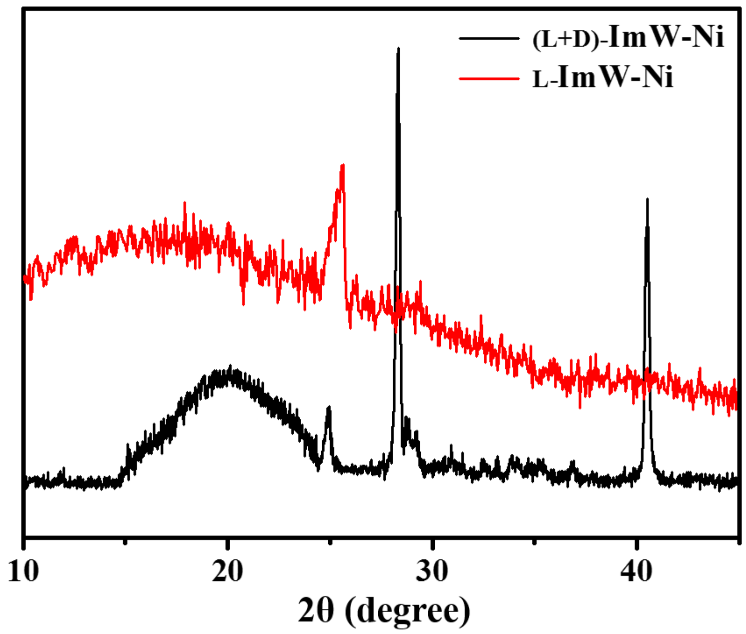

Fig. S15 XRD pattern of (L+D)-ImW-Ni xerogel and L-ImW-Ni powder.
